# Computer-generated dot maps as an epidemiologic tool: investigating an outbreak of toxoplasmosis.

**DOI:** 10.3201/eid0506.990613

**Published:** 1999

**Authors:** S B Eng, D H Werker, A S King, S A Marion, A Bell, J L Issac-Renton, G S Irwin, W R Bowie

**Affiliations:** Capital Regional District Health Department, Victoria, B.C; Canada. steven.eng@cephealth.org

## Abstract

We used computer-generated dot maps to examine the spatial distribution of 94 Toxoplasma gondii infections associated with an outbreak in British Columbia, Canada. The incidence among patients served by one water distribution system was 3.52 times that of patients served by other sources. Acute T. gondii infection among 3, 812 pregnant women was associated with the incriminated distribution system.


### Computer-Generated Dot Maps as an Epidemiologic Tool: Investigating an Outbreak of Toxoplasmosis

**Steven B. Eng,* Denise H. Werker,†‡ Arlene S. King,‡ Stephen A. Marion,§ Alison Bell,‡¶ Judith L. Issac-Renton,†§ G. Stewart Irwin,# and William R. Bowie§**

*Capital Regional District Health Department, Victoria, B.C., Canada; †Health Canada, Ottawa, Ontario, Canada; ‡British Columbia Centre for Disease Control, Vancouver, B.C., Canada; §University of British Columbia, Vancouver, B.C., Canada; ¶Centers for Disease Control and Prevention, Atlanta, Georgia, USA; and #Greater Victoria Water District, Victoria, B.C., Canada

We used computer-generated dot maps to examine the spatial distribution of 94 *Toxoplasma gondii* infections associated with an outbreak in British Columbia, Canada. The incidence among patients served by one water distribution system was 3.52 times that of patients served by other sources. Acute *T. gondii* infection among 3,812 pregnant women was associated with the incriminated distribution system.

Epidemiologists have traditionally used maps to examine the spatial distribution of disease incidence. Computer-generated dot maps have facilitated identification of case clusters (1), formulation of hypotheses about the source of infection or spatially distributed risk factors (2,3), and analysis of data. Because most populations are served by identifiable water systems, waterborne disease outbreaks lend themselves to being plotted on dot maps (1,4-6). We describe an automated address-matching and base map system in a geographic information system structure used to assess information related to an outbreak of toxoplasmosis associated with a municipal water system.

The Capital Regional District (population 321,585) is located on Vancouver Island, British Columbia, Canada. The district includes the City of Victoria, surrounding municipalities and districts, and the Gulf Islands.

In March 1995, an outbreak of toxoplasmosis was suspected when 15 residents of this district were identified as having acute infection with *Toxoplasma gondii*. Investigation of these and subsequent cases confirmed an outbreak but identified no common food, beverage, or event source. A hand-drawn dot map of the initial 47 cases showed clustering in the Greater Victoria area. The municipal water supply system was considered a possible explanation for this spatial distribution. To examine this hypothesis, computer-based geographic mapping was used to study the distribution of all outbreak-related acute cases and data collected from a population-based serologic screening program to detect *T. gondii* infection in women who were or had been pregnant (7).

Address for correspondence: Steven B. Eng, Health Protection and Environmental Services, Capital Health Region, #201-771 Vernon Avenue, Victoria, B.C., Canada, V8X 5A7; fax: 250-475-5130; e-mail: steven.eng@caphealth.org.

### Classification of Cases and the Water Distribution Systems

Patients were classified as having acute, equivocal, or nonacute cases or as never infected on the basis of serologic tests performed at the Provincial Laboratory (British Columbia Centre for Disease Control) and the Toxoplasma Serology Laboratory, Research Institute, Palo Alto Medical Foundation (8-11). Cases were further classified on the basis of clinical symptoms, outbreak relatedness, patient's residence, and pregnancy status (pregnant, nonpregnant) (7).

At the time of the outbreak, the Greater Victoria Water District operated two disinfection plants supplying unfiltered, chloraminated surface water to approximately 292,000 residents of the Capital Regional District. The higher-pressure Japan Gulch distribution system supplied water from the Japan Gulch Reservoir to 73,000 residents, as well as a one-way transfer of water into the other distribution system. The Humpback distribution system supplied 219,000 residents (12). District residents were further classified as receiving high, intermediate, or no exposure to water from the Humpback Reservoir.

### Geographic Mapping

Geographic mapping analysis was performed by using MapInfo Version 3.0 for Windows (MapInfo Corporation, Troy, NY, 1992-94). MapInfo, which enables data containing geographic information to be placed on a map, was used for geocoding (i.e., placing markers into the database, like pins onto a map). Street address data for each record in the datafile were matched against an electronic street map of the Capital Regional District. Geographic coordinates were taken from the electronic street map of the district, which is based on the Transportation Centerline Network of the British Columbia Ministry of Transportation and Highways (13). Lotus Approach 3.0 for Windows (Applied Software Corporation, subsidiary of Lotus Development Corporation) was used to prepare and validate the data before mapping. On the basis of information from the Greater Victoria Water District, a street-level scale map was drawn in MapInfo to delineate the geographic area in the Capital Regional District that receives municipal drinking water from the Humpback Reservoir.

### Geographic Distribution of Acute Cases

All 94 persons with outbreak-related acute cases who lived in the Capital Regional District were grouped by geographic area as being served by the Humpback Reservoir or other water sources. Incidence rates were calculated by using estimated population figures (1994). Information on residential addresses was obtained from laboratory requisitions, physicians' offices, hospital records, the Medical Services Plan of British Columbia, and direct communication with patients. For each case, the residential street address at the time the first specimen was drawn for testing for *T. gondii* antibodies was used to produce a computer-generated dot map.

Of the 94 persons with outbreak-related acute cases who lived in the Capital Regional District, 83 (88%) lived in the area served by the Humpback Reservoir. The incidence rate of acute infection among persons residing in the area served by the Humpback Reservoir was more than three times that for areas served by other sources (RR = 3.53; 95% confidence interval [CI]: 1.88-6.63; p = 0.0003) (Figure 1).

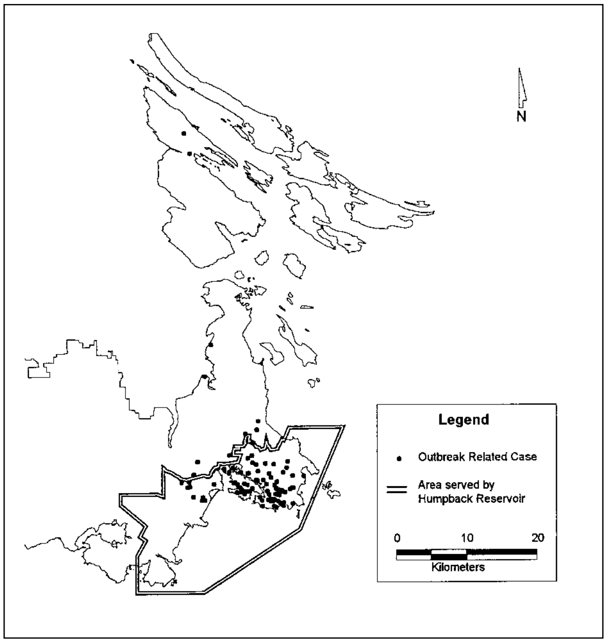

Figure 1. Geographic distribution of outbreak-related acute cases of toxoplasmosis in the Capital Regional District, Vancouver Island, British Columbia, 1995 (n = 94).

### Geographic Distribution of Women Screened during Pregnancy

Data from a population-based screening program were used to determine whether residents served by the Humpback Reservoir were more likely to have acute infection with *T. gondii*. Serologic screening was offered to an estimated 4,500 women living in the capital regional district who were pregnant between October 1, 1994, and April 30, 1995. To offer screening to as many of these women as possible, information regarding the screening program was extensively distributed to women, physicians, and the public.

Serologic results were available from the Provincial Laboratory database at the British Columbia Centre for Disease Control. Residential street addresses were obtained by linking the Provincial Laboratory database with the Medical Services Plan database, using the unique personal health number assigned by the Province. A computer datafile containing the serologic results of screened pregnant women and their addresses was provided to the Capital Regional District Health Department. The data were then prepared and validated, geocoded and mapped, and statistically analyzed.

Three dot maps were generated by MapInfo, showing the geographic distribution of the screened population according to their laboratory classification of never infected (i.e., no serologic evidence of immunoglobulin [Ig] G or IgM antibody to *T. gondii*), nonacute (i.e., typically IgG but not IgM antibody to *T. gondii*), and acute (i.e., serologic evidence of acute infection by a battery of tests) (7-11). Several data subsets were generated on the basis of two variables: 1) residence in the area served by the Humpback Reservoir and 2) municipality of residence. Odds ratios were then calculated by StatCalc in Epi Info (version 6.03) to test the hypothesis that living in the area served by the Humpback Reservoir was associated with infection with *T. gondii*.

Of 3,982 laboratory records for screened women, 3,962 records were available for coding. The 3,812 women with successfully coded addresses comprise 85% of the estimated 4,500 women eligible for screening. Of these women, 36 (0.9%) were classified according to their laboratory results as having acute infection, 216 (5.7%) as having a nonacute infection, 3,558 (93.3%) as never having been infected, and 2 (0.1%) as having equivocal cases. The age distribution of the women (15 to 45 years [mean 29 years]) did not differ by infection status (women with equivocal status were excluded). Women acutely infected with *T. gondii* were more than three times as likely as uninfected women to live in the area served by the Humpback distribution system (odds ratio [OR] 3.05, exact 95% CI: 1.08-11.91). In contrast, the geographic distribution of women with serologic evidence of nonacute infection with *T. gondii* was not associated with the municipal water distribution system (OR 0.91, exact 95% CI: 0.67-1.25).

Computer-generated dot maps (Figures 2, 3, 4) provided visual information, as well as data for

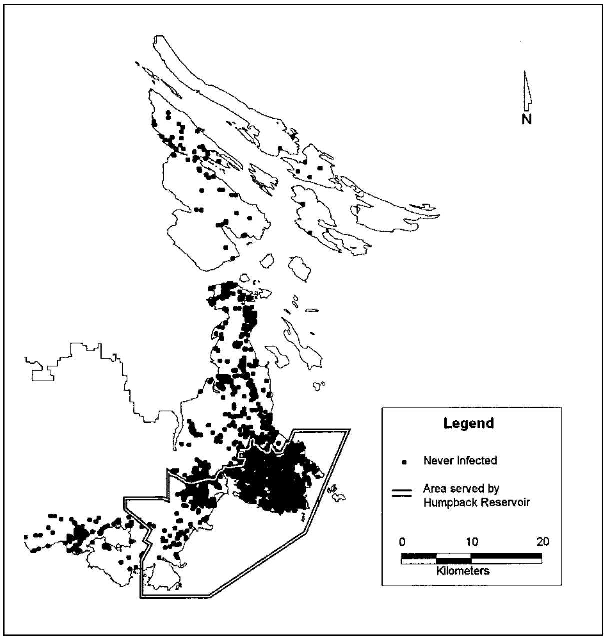

Figure 2. Geographic distribution of the residences of women screened during pregnancy and classified as never infected with toxoplasmosis (n = 3558).

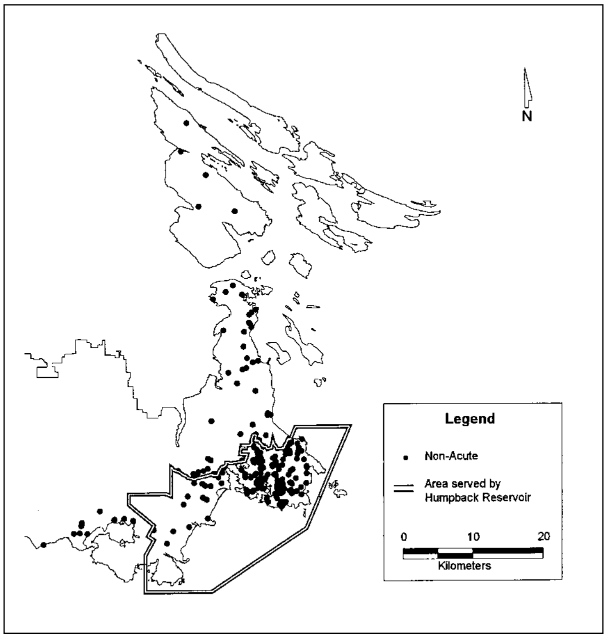

Figure 3. Geographic distribution of the residences of women screened during pregnancy and classified as having nonacute cases of toxoplasmosis (n = 216).

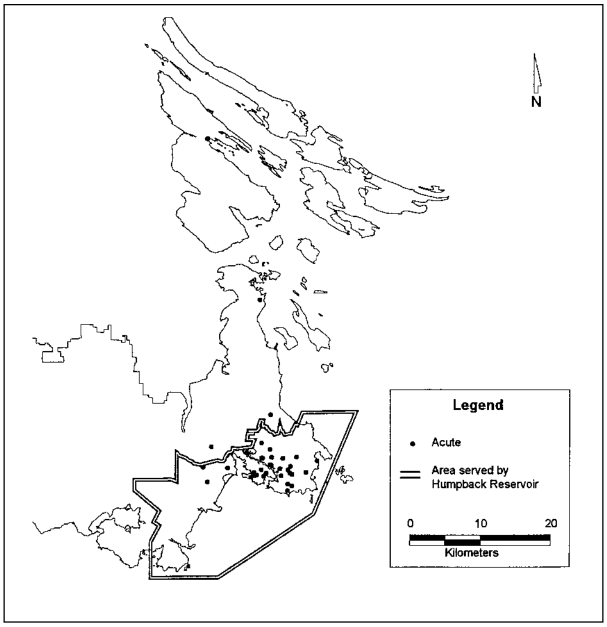

Figure 4. Geographic distribution of the residences of women screened during pregnancy and classified as having acute cases of toxoplasmosis (n = 36).

statistical analysis. These maps are characteristic of an outbreak associated with a one-time event. The geographic distribution of pregnant women who were never infected (Figure 2) indicates that the susceptible population is similar to the distribution of the whole population. The geographic distribution of pregnant women who had nonacute cases (Figure 3) is similar to the population distribution, indicating that before the outbreak, residence was not associated with antibody. Visually comparing the geographic distribution of pregnant women with acute cases (Figure 4) with that of pregnant women who were never infected (Figure 2) suggests that most of the recently infected pregnant women lived in the area served by the Humpback Reservoir. The difference between the distributions seen in Figures 3 and 4 suggests that this event was new and unusual, rather than an ongoing or intermittent exposure that had not been recognized.

Well-defined geographic areas of the Capital Regional District were classified as receiving high, intermediate, and no exposure to water from the Humpback Reservoir (exposure scores 3, 2, and 1, respectively). An analysis for linear trend in proportions was performed by using StatCalc in Epi Info to demonstrate whether a dose-response relationship was present.

If water from the Humpback Reservoir was the source of infection, the rate of infection would be expected to increase with increased exposure. The Table shows the geographic distribution of women according to the estimated concentration of water from Humpback Reservoir received at their residence. When acutely infected women were compared with seronegative women, the trend in linear proportions across the three exposure scores was significant (chi-square = 6.67; p = 0.01). In contrast, a significant linear trend in proportions was not demonstrated when women with nonacute cases were compared with seronegative women (chi-square = 1.30; p = 0.25).

### Conclusions

The conclusions drawn from geographic mapping depend on the accuracy and validity of the datasets and the availability of denominator data to avoid making false associations that are a function of population densities. For the overall population, denominators were available from census data. Firm denominator data were available from the serologic screening study.

With respect to accurate placement on the maps, the addresses and the delineation of the water distribution systems were critical to the evaluation. Although extensive efforts were

Table. Linear trend analysis of acute and nonacute infections with *Toxoplasma gondii* among screened pregnant women, by degree of exposure to Humpback Reservoir water

| | Rank of exposure | Exposure score | Infected (cases) | Never infected (controls) | Odds ratio (relative to baseline) | Trend test p value[a] |
|---|---|---|---|---|---|---|
| Acute | No | 1 | 4 | 983 | 1.00 | |
| (recent | Intermediate | 2 | 10 | 1,078 | 2.28 | |
| infection) | High | 3 | 22 | 1,497 | 3.61 | 0.01 |
| Nonacute | No | 1 | 64 | 983 | 1.00 | |
| (past | Intermediate | 2 | 71 | 1,078 | 1.01 | |
| infection) | High | 3 | 81 | 1,497 | 0.83 | 0.25 |

[a]Extended Mantel-Haenszel chi-square, Schlesselmann JJ. Case-control studies, New York, Oxford University Press, 1982.

made to verify addresses, there was some potential for misclassifying exposure. However, any misclassification resulting from the use of the Medical Services Plan database should not have systematically biased our analyses.

The Greater Victoria Water District engineers delineated the boundaries of the Humpback distribution system and ranked the zones in the Capital Regional District according to the proportion of water received from the Humpback Reservoir. As the engineers had no prior knowledge of the distribution of outbreak-related cases of toxoplasmosis, it is unlikely that the determination of these geographic boundaries was biased.

A limitation of the geographic mapping analysis of cases is that rates were not adjusted for age or other covariates. However, the subsequent analyses using data from the population-based screening of pregnant women were not confounded by age. The age distribution of the women in these analyses did not differ when grouped by infection status.

Computer mapping software had the advantages of 1) facilitating the verification and correct placement of addresses, 2) reducing the time required to map the location of large datasets, 3) enabling queries and statistical analysis of the data after mapping, 4) allowing several sets of mapped data to be analysed simultaneously for potential relationships, and 5) generating printouts or overheads for presentations.

### Acknowledgments

We thank Timothy Johnstone, Louise Egan, Michael Aeberhardt, Kevin Kirkwood, Bill Keenan, and Jenny Cadman for invaluable technical assistance and critical information. We also thank Jack S. Remington and the Toxoplasma Serology Laboratory, Research Institute, Palo Alto Medical Foundation.

This investigation was supported by the Ministry of Health, British Columbia; the Capital Regional District Health Department, Victoria, British Columbia; and the Laboratory Centre for Disease Control, Health Canada, Ottawa, Ontario.

Mr. Eng is with the Health Protection and Environmental Program for Greater Victoria, British Columbia. His interests focus on environmental epidemiology, manipulation and analysis of computer data, and computer-based mapping.

### References

1. Barreto ML. The dot map as an epidemiological tool: a case study of *Schistosoma mansoni* infection in an urban setting. Int J Epidemiol 1993;22:731-41.
2. Glass GE, Schwartz BS, Morgan JM, Johnson DT, Noy PM, Israel E. Environmental risk factors for Lyme disease identified with geographic information system. Am J Public Health 1995;85:944-8.
3. Skorpanich MA, Taylor TH, Anton-Culver H. Mapping disease and risk factors to identify public health concerns. In: URISA 88. Urban & Regional Information Systems Association, Los Angeles, CA 1988;4:310-9.
4. Timmreck TC. An introduction to epidemiology. Boston: Jones and Bartlett Publishers, Inc.; 1994.
5. Rosenberg ML, Haziet KI, Schaefer J, Wells JG, Pruneda RC. Shigellosis from swimming. JAMA 1976;236:1849-52.
6. Merson MH, Goldmann DA, Boyer KM, Peterson NJ, Patton C, Everett LG, et al. An outbreak of *Shigella sonnei* gastroenteritis on Colorado River raft trips. Am J Epidemiol 1974;100:186-95.
7. Bowie WR, King AS, Werker DH, Issac-Renton JL, Bell A, Eng SB, et al. Outbreak of toxoplasmosis associated with municipal drinking water. Lancet 1997;350:173-7.
8. Remington JS, McLeod R, Desmonts G. Toxoplasmosis. In: Remington JS, Klein JO, editors. Infectious diseases of the fetus and newborn. 4th ed. Philadelphia (PA): W.B. Saunders Company; 1995. p. 140-267.
9. Wong S-Y, Remington JS. State of the art clinical article: Toxoplasmosis in pregnancy. Clin Infect Dis 1994;18:853-62.
10. Montoya JG, Remington JS. Studies on the serodiagnosis of toxoplasmic lymphadenitis. Clin Infect Dis 1995;20:781-9.
11. Dannemann BR, Vaughan WC, Thulliez P, Remington JS. Differential agglutination test for diagnosis of recently acquired infection with *T. gondii*. J Clin Microbiol 1990;28:1928-33.
12. Irwin GS, Zanette M, Morris B. Greater Victoria's drinking water system 1994 bacteriological summary. Greater Victoria Water District, Victoria, British Columbia, Canada; 1995.
13. British Columbia Roads and Rivers (TcN data), based on Transportation Centerline Network (1993), Planning Services Branch, Ministry of Transportation and Highways, Province of British Columbia.

